# Unexpected Cardiogenic Shock in a Young Woman: A Case Report

**DOI:** 10.1155/cric/7627691

**Published:** 2026-10-02

**Authors:** Benjamin A. Johnson, Luis A. Ortiz, Xavier E. Prida

**Affiliations:** ^1^ Department of Internal Medicine University of South Florida Tampa Florida USA usf.edu; ^2^ Department of Internal Medicine Division of Cardiovascular Sciences University of South Florida Tampa Florida USA usf.edu

## Abstract

Recent cardiogenic shock (CS) literature emphasizes early recognition, staging, and treatment to improve survival, highlighting the need for system‐wide detection pathways and prompt shock team activation to enable timely intervention. We present a 26‐year‐old woman initially diagnosed with septic shock who deteriorated despite therapy. Ongoing reassessment led to reclassification as CS, prompting shock team activation and initiation of mechanical circulatory support, ultimately resulting in full recovery. CS is uncommon in young adults and may be misdiagnosed as distributive shock. Repeated evaluations with focused examinations, laboratory testing, and echocardiography are critical for early detection and proper diagnosis and management.

## 1. Introduction

Cardiogenic shock (CS) is a state of end‐organ hypoperfusion due to cardiac failure and primarily affects an older population with underlying cardiac risk factors [1, 2]. Over the past decade, the prevalence of CS from acute myocardial infarction has decreased, whereas the proportion of CS from other causes like acute decompensated heart failure has increased [3]. CS is more likely to be of low clinical suspicion in the young patient population because of its rare prevalence and low rates of acute myocardial infarction and chronic heart failure in this demographic, leading to delays in time‐critical care. The most recent CS literature, including the DanGer‐Shock trial, emphasizes that early recognition, staging, and treatment of CS is crucial to improving survival and, subsequently, highlights the need for system‐wide detection pathways and prompt shock team activation to enable timely intervention [4]. Repeated evaluation with focused examination, laboratory testing, and echocardiography is also critical for early detection. This case report illustrates a reversible CS phenotype and reinforces the importance of multidisciplinary shock teams and structured activation protocols.

## 2. Case Presentation

A 26‐year‐old female with a past medical history of Class III obesity initially presented to an urgent care facility and then was sent to the Emergency Department for a worsening cough and dyspnea over the last few days. In the week leading up to this presentation, symptoms of cough, white sputum production, lower extremity swelling, and orthopnea were reported, which improved with over‐the‐counter medications. There was no recent travel, fevers, chills, drug use, sick contacts, nausea, vomiting, or diarrhea. On the initial exam, vitals showed a temperature of 98.8°F, heart rate of 140 bpm, respiratory rate of 28 breaths/min, and blood pressure of 145/102 mmHg. She was ill‐appearing, with trace bilateral pedal edema, warm extremities, clear lungs on auscultation, no appreciable JVD, and normal mentation. Initial differential diagnoses based on her presentation and age included sepsis secondary to bacterial pneumonia, pulmonary embolism, heart failure, pneumothorax, and anemia. Labs on admission revealed an elevated WBC count (16.52 × 10^9^/L), AST (64 U/L) and ALT (118 U/L), BNP (651 pg/mL), HS‐troponin (34 ng/L), CRP (1.76 mg/dL), and procalcitonin (0.13 ng/mL). The Urine *Streptococcus pneumoniae* antigen was positive. A chest X‐ray did not show any acute abnormalities. The ECG showed sinus tachycardia with poor R wave progression (Figure 1). A CT angiography of the pulmonary arteries did not show any evidence of pulmonary embolism, but diffuse, bilateral, multifocal ground‐glass densities were described, most prominent at the lung bases with prominent mediastinal and hilar lymph nodes. A right upper quadrant ultrasound showed moderate to severe hepatic steatosis.

**Figure 1:**
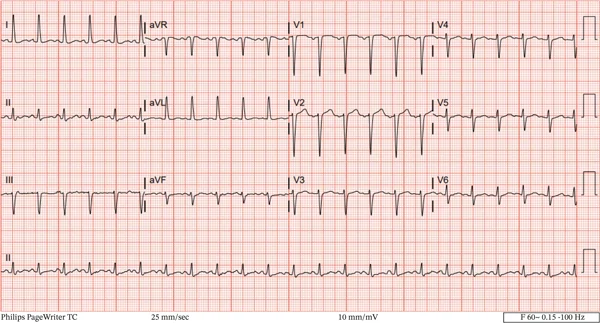
ECG demonstrating sinus tachycardia with poor R wave progression.

The patient was admitted to the medical ICU for treatment and monitoring of presumed sepsis and acute hypoxic respiratory failure suspected secondary to community acquired pneumonia with institution of empiric, broad spectrum antibiotics and BiPAP ventilation support. On hospital Day 2, an echocardiogram was obtained showing an ejection fraction (EF) of 30%–35% with global hypokinesis, mild to moderate mitral valve regurgitation, and normal right ventricular systolic function (Figure 2). On the same day, progressive respiratory failure attributed to ARDS from *S. pneumoniae* required endotracheal intubation and mechanical ventilation. On hospital Day 4, hypotension and oliguria developed, which were primarily attributed to septic shock and required vasopressor support. However, because of the patient′s declining status and reduced EF, the critical care team turned their diagnostic and treatment strategy toward CS and a bedside pulmonary artery catheter (PAC) was placed.

**Figure 2:**
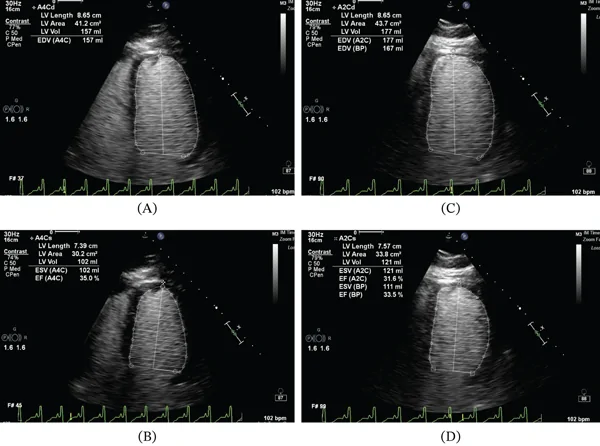
Transthoracic echocardiogram ejection fraction (EF) by Simpson′s biplane method. (A) Four‐chamber diastolic view. (B) Four‐chamber systolic view. (C) Two‐chamber diastolic view. (D) Two‐chamber systolic view.

The initial hemodynamic measurements based on the bedside PAC while the patient was temporarily off of any vasopressor support revealed a central venous pressure (CVP) of 23 mmHg, pulmonary artery pressure (PAP) of 62/40 mmHg, pulmonary capillary wedge pressure (PCWP) of 40 mmHg, and cardiac index (CI) of 1.87 L/min/m^2^ with a systemic vascular resistance (SVR) of 1034 dynes.sec/cm^−5^. On hospital Day 4, the Cardiogenic Shock (C‐SHOCK) team—which is a multidisciplinary team available 24 hours a day, 7 days a week, comprised of attending physicians from Heart Failure Cardiology, Interventional Cardiology, Cardiothoracic Surgery, and Critical Care Medicine was consulted with transfer of the patient to the Cardiac ICU for optimal care. Because of the low CI and severely elevated wedge pressure, inotropic support with intravenous milrinone and diuresis with continuous bumetanide infusion was begun. The etiology of the new cardiomyopathy was unclear, so on hospital Day 6, the Advanced Heart Failure team completed an endomyocardial biopsy with a right heart catheterization from the left femoral vein which was 2 days after the initial bedside left jugular vein PAC placement and aggressive diuresis. The study revealed elevated filling pressures and mild, combined pre‐ and postcapillary pulmonary hypertension with a mean PAP of 34 mmHg, PCWP of 14 mmHg, transpulmonary gradient (TPG) of 20 mmHg, diastolic pulmonary gradient (DPG) of 11 mmHg, pulmonary vascular resistance (PVR) by thermodilution of 4.1WU, cardiac output (CO) of 5.3 L/min, CI of 2.4 L/min/m^2^, PVR to SVR ratio of 0.3, and Pulmonary Artery Pulsatility Index (PAPi) of 2.4. During this study, the patient was receiving milrinone 0.375mcg/kg/min, vasopressin 0.02 units/min, and bumetanide 0.5 mg/h. On hospital Day 7, the patient′s oxygenation worsened despite ongoing therapy. Based on the composite data, the C‐SHOCK team recommended and implemented VV ECMO and microaxial flow pump (mAFP) support with an Impella CP to unload the left ventricle.

On hospital Day 7, the endomyocardial biopsy results showed inconclusive evidence of myocarditis because of the absence of unequivocal myocyte injury caused by the inflammatory infiltrates observed. On hospital Day 10, because of worsening LV and RV function, a step‐up circulatory support occurred with the exchange of the Impella CP for an Impella 5.5. On hospital Day 12, the VV ECMO circuit was removed as the patient′s oxygenation continued to improve. On hospital Day 17, the Impella 5.5 was removed because of partial LV function recovery noted by improving CI with weaning of the mAFP flow. Following tracheostomy on hospital Day 19 for prolonged need for mechanical ventilation, the patient′s respiratory status slowly improved and mechanical ventilation was eventually completely discontinued. The patient was ultimately discharged home on hospital Day 40. A cardiac MRI completed 1 day prior to discharge demonstrated a recovered EF of 53% with no regional wall motion abnormalities and no late gadolinium enhancement.

During a follow‐up appointment 2 months after discharge with the Advanced Heart Failure team, the patient was doing well and working from home as a YouTube video editor.

## 3. Discussion

This patient case emphasizes the importance of continued surveillance and reclassification of CS particularly in young patients with atypical presentation. Once reclassified, syndrome specific management by a designated team allowing for the escalation of life‐saving therapies occurred. Our patient, a 26‐year‐old woman with Class III obesity, presented with progressive dyspnea, orthopnea, peripheral edema, cough, hypoxia, and eventual hypotension requiring vasopressors—a clinical picture that initially led to a presumed diagnosis of pneumonia and septic shock. Rapid clinical deterioration despite appropriate sepsis therapy highlighted the need for further clinical evaluation and led to the identification of a low CO, which likely represented a mixed vasodilatory‐CS, possibly due to sepsis‐induced cardiomyopathy or myocarditis [5, 6].

Early identification of CS is critical, as delays in diagnosis contribute to poor outcomes. The Society for Cardiovascular Angiography and Interventions (SCAI) shock classification system stratifies patients based on severity and guides treatment escalation [7–9]. On initial encounter, our patient had a clinical syndrome that should raise suspicion for early (SCAI Stage B) CS. The 2022 AHA/ACC/HFSA heart failure guidelines recommend early echocardiography and invasive hemodynamic assessment in patients with suspected CS, especially when clinical presentation is unclear [10]. These tools, combined with biomarker data (lactic acid, NT‐pro BNP, HS‐troponin, creatinine, ALT, and ABG or VBG) and clinical features (altered mental status, cool extremities, weak pulses, elevated JVP, orthopnea, lower extremity edema, and tachycardia), are essential to distinguishing CS from septic shock or primary pulmonary pathology [11, 12]. Current literature supports the importance of early and repetitive measurement of lactic acid in patients across the continuum of SCAI stages of CS. Both an initially higher lactic acid and slower lactic acid clearance have been associated with worse mortality [13]. Clinical criteria for CS include sustained hypotension defined as a systolic blood pressure < 90 mmHg or mean arterial pressure < 60–65 mmHg for at least 30 min with evidence end‐organ hypoperfusion shown commonly by a serum lactate > 2 mmol/L, oliguria, or elevated liver enzymes, as well as other signs such as altered mental status and cool extremities and is often accompanied by a heart rate > 100 bpm. In the case presented, an earlier bedside or formal echocardiography may have led to early detection of LV dysfunction and increased clinical suspicion for CS.

Unlike distributive (sepsis) and hypovolemic shock—where structured early warning systems with validated scoring tools such as SIRS, qSOFA, and lactate levels, and rapid‐response protocols are routinely applied—CS lacks equally standardized and utilized early detection tools and clinical triggers for care escalation. This discrepancy often leads to delays in diagnosis and intervention, particularly in normotensive patients with compensated physiology. Our case highlights the importance of maintaining a high degree of clinical suspicion for CS with refining of differential diagnoses based on ongoing data accumulation. Developing formalized pathways for early CS recognition and integrating them into emergency, wards, and ICU workflows may help close this gap and enable earlier activation of shock teams, which has been shown to improve outcomes [14].

By the time of intubation and vasopressor initiation, our patient met criteria for SCAI Stage C shock and invasive hemodynamic evaluation showed elevated filling pressures (PCWP 40 mmHg), low CI (1.87 L/min/m^2^), and systemic congestion. Notably, her initial normotension, warm extremities, and preserved mentation initially masked the severity of her cardiac dysfunction—a scenario increasingly recognized in younger patients with robust compensatory mechanisms [9]. SCAI stage reclassification during hospitalization is a particularly potent predictor of in‐hospital mortality especially in the first 24 h of admission as demonstrated by the Altshock‐2 registry [15].

Once CS was identified, activation of our institution′s C‐SHOCK team enabled escalation to inotropic support, IV diuresis, and mAFP support. Early use of mAFP support is consistent with emerging consensus favoring early rather than delayed device deployment in deteriorating patients [9, 10]. Importantly, the patient experienced complete recovery of left ventricular function and was successfully weaned off all mechanical support within 17 days.

Although the endomyocardial biopsy was not diagnostic for myocarditis, the clinical course was consistent with a reversible cardiomyopathy. Follow‐up cardiac MRI demonstrated normalized systolic function and no evidence of delayed gadolinium enhancement, supporting a nonischemic, nonfibrotic etiology.

## 4. Conclusions

We present a case of unexpected CS in a young woman highlighting the importance of maintaining a high level of clinical suspicion for CS with appropriate and timely management and frequent SCAI reclassification (Figure 3). The multidisciplinary approach demonstrated by the C‐SHOCK team allowed for efficient step‐up and step‐down of advanced circulatory support therapies which was critical for our patient′s successful clinical course. This case study demonstrates the need for consistent collection of the initial data set to raise suspicion of CS and presents an opportunity for clinical pathways or order sets for CS workup and management to be developed in order improve outcomes.

**Figure 3:**
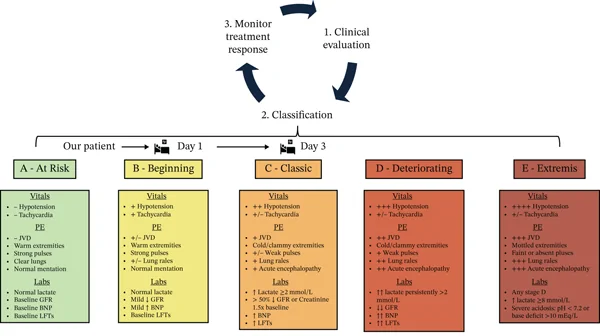
Cardiogenic shock evaluation and reclassification schema.

## Funding

No funding was received for this manuscript.

## Ethics Statement

Written informed consent was obtained from the patient for publication of this case report and any accompanying images. Institutional Review Board approval was not required for this case report in accordance with local institutional policies. All identifying information has been removed to protect patient confidentiality.

## Conflicts of Interest

The authors declare no conflicts of interest.

## Data Availability Statement

The data that support the findings of this study are available on request from the corresponding author. The data are not publicly available due to privacy or ethical restrictions.
